# Sweetening fear exposure: study protocol for a multi-day, randomized controlled trial to study effects of glucose in exposure sessions of participants with public speaking anxiety

**DOI:** 10.1186/s40359-026-04125-0

**Published:** 2026-02-18

**Authors:** Monika Lehnert, Tanja Michael, Lea Berger, Alexander Hauck, Diana S. Ferreira de Sá

**Affiliations:** Department of Psychology, Division of Clinical Psychology and Psychotherapy, Saarland University, Saarbrücken, Germany

**Keywords:** Public Speaking Anxiety, Exposure Therapy, Social Anxiety Disorder, Glucose, Psychotherapy Adjuvants

## Abstract

**Background:**

Public speaking anxiety (PSA) is present in most individuals with social anxiety disorder (SAD). As for SAD, exposure therapy is considered the gold-standard treatment for PSA. However, it is known that not all patients benefit from it. Recent research (Hauck Behav Res Ther: 104553, 2024) suggests that glucose facilitates fear extinction and consolidation, processes crucial for psychotherapeutic success, demonstrating its potential as an adjuvant to improve the efficacy of exposure therapy. The present study aims to translate previous findings into a clinical context, by studying the effects of glucose on fear exposure for participants with PSA.

**Methods:**

Participants with PSA will complete a spaced multi-day public speaking exposure procedure (Cheng J Behav Ther Exp Psychiatry: 54;101-107, 2017), an established and successful method of investigating treatment for PSA (Culver J Behav Ther Exp Psychiatry: 43:787-93, 2012; Niles Behav Res Ther: 68:27-36, 2015). The effectiveness of the exposure treatment will be measured through self-report assessment and the physiological measures skin conductance (SC), and heart rate (HR). Participants will be randomly assigned to receive either glucose or a placebo prior to exposure in a randomized controlled trial.

**Discussion:**

This study is the first to investigate glucose administration as cognitive enhancer in a subclinical population, emphasizing the transition from bench to bedside. We provide a detailed study protocol to increase transparency in open science as well as enabling researchers to use the detailed protocol to implement with other manipulations, to improve exposure therapy in the long run.

**Supplementary Information:**

The online version contains supplementary material available at 10.1186/s40359-026-04125-0.

## Background

Anxiety disorders (AD) represent the most prevalent category of mental health disorders, affecting around 4.4% of the global population [1]. Over a time period from 1990–2019 the incidence of ADs increased globally [2]. Despite strong evidence for the efficacy of Cognitive Behavioral Therapy (CBT) and exposure therapy as indicated treatment for ADs, treatment outcomes are not equally beneficial for all patients [3–5]. Indeed, response rates of patients indicate that only around half of patients achieve clinically meaningful gains, both at the end of treatment and during follow-up assessments, emphasizing the importance of continued research on mechanisms and novel interventions [6]. Jorm and colleagues [7] investigated therapy outcomes in high-income countries and found that, despite increased amount of therapy, prevalence rates are not decreasing. They concluded that the implemented therapy also needs to improve in terms of standardization. To improve exposure therapy and to effectively investigate possible adjuvants, this study protocol aims to provide a standardized framework for following research on adjuvants in exposure treatments. Considering the need to enhance the effectiveness of exposure therapy, public speaking anxiety (PSA) offers an ideal alternative for experimental research to investigate therapeutic mechanisms without the ethical and logistical challenges of working directly with clinical patients. Classified as a specific subtype of SAD [8, 9], PSA is defined as the fear of speaking in front of an audience [9, 10]. Individuals affected by PSA experience strong physiological and cognitive symptoms of fear, such as trembling or shaking, blushing, a racing heart, feelings of nervousness and tension, and negative thoughts when they are required to speak in front of an audience [9, 11]. PSA was found to be the most common social fear with a prevalence rate of 70.3% in patients with SAD [9, 12]. PSA is associated with substantial distress and functional impairment in social, academic, and occupational domains, largely due to heightened psychological and physiological reactivity in performance situations [9]. A dominant theoretical framework for understanding ADs builds on fear conditioning models [13–15]. In these models, a previously neutral stimulus acquires threat value through association with an aversive event, leading it to trigger fear responses. The idea behind fear exposure is to create a new association pattern, where the feared object is linked to the absence of a threat, and thus with an absence of fear. This new association competes with the old one and the more this new association is consolidated, the more dominant it becomes in recall compared to the original fear association [16]. To improve the consolidation of this so-called extinction memory, several cognitive enhancers like cortisol [17–20], insulin [21], D-cycloserine [22–24], among others, have been investigated with promising results. A previous study found that healthy participants receiving insulin, which largely affects the glucose metabolism, showed significantly less fear responses during fear extinction and a better extinction recall in comparison to a control group [21]. Glucose has been shown before to be a potential cognitive enhancer with studies showing a facilitated cognitive functioning in general and especially in episodic memory and working memory in healthy participants [25–29], while being easily and safely administered through oral ingestion, with no significant limitations or side effects [29–31]. Glucose consumption after fear learning increases hippocampal-dependent contextual fear conditioning processes [32]. More recently, Hauck and colleagues (2024) showed that glucose administration before extinction learning accelerates the extinction of acquired fear in healthy participants, while glucose administration after extinction learning was associated with an earlier consolidation of extinction memory [31]. As these studies primarily used fear-conditioning paradigms with healthy participants, it is crucial to extend this research into clinical context in order to enhance the translational value of the findings for improving exposure therapy. With this in mind, the present study investigates whether glucose might have an impact on the learning processes that take place during fear exposure in participants with PSA. As exposure intervention for PSA, the paradigm from Cheng and colleagues (2017) was adapted to fit the manipulation with an administration of glucose. This paradigm has been widely used to investigate mechanisms underlying exposure therapy, particularly in socially anxious populations [11, 33, 34].

## Objective of this study

Following previous results of glucose enhancement of fear extinction processes, the present study aims to investigate if the same can be translated into a more clinical context. For that purpose, participants with PSA will be randomly assigned to an intervention group (Glucose vs Placebo) and will accordingly receive an equally tasting [28] glucose or placebo drink before starting with an exposure protocol (session 1 and 2). The participants will complete an online pretest, 3 days of experiment (session 1–3) and a follow-up. The first two sessions are spaced by 2 days, and the last two sessions are spaced by 5 days. On both sessions 1 and 2 participants complete a behavioral approach test (BAT) and full exposure protocol, and on session 3 participants only complete a BAT. For the pretest and the follow-up participants receive a link to complete self-rated questionnaires. Further information can be found in the procedure. The following hypotheses will be tested:

- (H1) Participants in the glucose group show a greater decline in self-report ratings (Subjective Units of Disturbance Scale (SUDS)) in the Behavioral Approach Test (BAT) in sessions 2 and 3 (vs. session 1) compared to the placebo group.
- (H2) Participants in the glucose group show a greater decline in Personal Report of Public Speaking Anxiety (PRPSA) ratings in sessions 2, 3 (vs. session 1) compared to the placebo group.
- (H3) Participants in the glucose group show a greater decline in self-report ratings (SUDS) towards the end of the exposure sessions (on sessions 1 and 2) compared to the placebo group.
- (H4) A similar pattern to H1 and H2 should be seen regarding the physiological measures SC and HR.
- (H5) Participants in the glucose group show a greater gain from the exposure sessions at follow-up regarding self-report measures compared to the placebo group.

The present study is pre-registered on the Open Science Framework (OSF) and will remain under embargo until study completion (https://osf.io/bng5z) [35].

## Methods

### Participants

Participants are recruited via Flyers, distributed at the university, in the surrounding area and via social media platforms. Participants will receive either €60 or 6 course credits upon completing the study (the last only applicable for Psychology students at the Saarland University). In cases of dropout/discontinued participation, compensation will be adjusted proportionally to the extent of participation.

### Harms

Based on previous studies using similar paradigms, the procedures used in this study are considered safe and are not expected to result in adverse effects. Contact information for the Department of Psychotherapy at Saarland University will be provided in the unlikely event that participants experience prolonged discomfort or distress.

### Eligibility criteria

For inclusion, participants must be of legal age. Participants with a history of psychological disorders are excluded from participation. However, individuals meeting criteria for SAD will be eligible for inclusion, since this is a common diagnosis associated with PSA. To fulfill the inclusion criteria of PSA, participants are evaluated using two screening questions [11, 34, 36]. A value of 6 or higher on anxiety (“How anxious would you feel giving a formal speech in front of a live audience?”) and 5 or higher on avoidance (“How likely would you be to avoid taking a class that required an oral presentation?”) leads to inclusion. Responses are given on a 0–8 scale, where 0 indicates “no anxiety/never avoid” and 8 indicates “extreme anxiety/always avoid”. Individuals with any acute or chronic medical condition (e.g., diabetes, epilepsy, cardiac pacemakers) are not eligible for inclusion. Only individuals with a body mass index (BMI) within the normal range (18.5–26) are included. Regular intake of medication leads to exclusion, except for oral contraceptives. Additional exclusion criteria include drug use, defined as regular consumption within the previous six months.

### Sample size

The planned sample size was determined using an exact simulation-based power analysis tool [37]. To test the main hypotheses, an interaction effect of a 2 × 3 Mixed-Design ANOVA, a sample size of N = 76 (38 per group) was estimated, based on an alpha level of 0.05 and a power of 0.80.

### Materials and measures

In the following, all measurements relevant to the main hypothesis are presented. Additional questionnaires and measures administered in the study can be found in supplementary file 1.

### Subjective measures / self-report measures

#### Personal report of public speaking anxiety (PRPSA)

The PRPSA [38] assesses PSA using 34 items rated on a Likert scale from 1 (*strongly disagree*) to 5 (*strongly agree*). Total scores range from 34 to 170, with higher values indicating greater anxiety.

#### Self Statements during Public Speaking Scale (SSPS)

The SSPS [39],German Version: [40] assesses cognitive aspects of public speaking using ten items rated from 0 (*do not agree at all*) to 5 (*extremely agree*). It provides separate scores for positive and negative thoughts (0–25 each) and demonstrates good psychometric properties.

#### Scales for social anxiety disorders (Sozas)

The SOZAS [41] include four measures of social phobia,the present study used three of them: 1) the Social Phobia Inventory (SPIN, [41, 42]), 2) the Social Phobia Scale, and 3) the Social Interaction Anxiety Scale (SPS, SIAS, [43],German Version: [44]). The SPIN comprises 17 items rated from 0 (*none*) to 4 (*extreme*), with total scores ranging from 0 to 68; values above 25 indicate the presence of social phobia. The SPS and SIAS each include 20 items rated from 0 (*strongly disagree*) to 4 (*extremely agree*). Cutoff scores > 17 (SPS) and > 26 (SIAS) distinguish individuals with social phobia from healthy controls. All three instruments demonstrate good to excellent psychometric properties.

#### Subjective units of distress scale (SUDS)

The SUDS is a one-item 11-point Likert-type subjective anxiety scale. It measures the self-rated current anxiety on a scale from 0 (*totally relaxed*) to 100 (*highest anxiety ever felt*). The midpoint “50” indicates “Moderate anxiety, but able to continue” [45, 46]. The scale is also referred to as Subjective Units of Disturbance Scale.

#### Generalized anxiety disorder (GAD-7)

The GAD-7 [47],German version: assesses generalized anxiety symptoms using seven items rated from 0 (*not at all*) to 3 (*nearly every day*). Total scores range from 0 to 21, with cut-offs indicating minimal (0–4), mild (5–9), moderate (10–14), and severe (15–21) anxiety. The GAD-7 demonstrates good sensitivity and is a reliable screener for anxiety disorders, including social anxiety [48].

#### Patient health questionnaire (Depression and Stress Scales) (PHQ-9)

The PHQ-9 Depression [49],German version: [48] assesses depression with nine items on a scale from 0 (*not at all*) to 3 (*almost every day*). A total score is formed by adding up all items. Scores under five indicate “healthy”, 5–10 “unremarkable”, 10–15 “mild depression”, 15–20 “moderate depression”, and 20–27 “severe depression” [48]. The PHQ Stress Scale consists of ten items, which are measured on a scale ranging from 1 (*not impaired*) to 3 (*heavily impaired*). A total score is formed by adding up all items. Higher scores indicate stronger impairment [48].

#### Depression Anxiety Stress Scale (DASS-21)

The DASS-21 [50],German version: [51] is used to assess depressive symptoms. The scale has 21 items ranging from 0 (*did not apply to me at all*) to 3 (*applied to me very much or most of the time*). The total score ranges from 0 to 63. Higher scores indicate higher levels of depressive symptoms. The questionnaire demonstrated good psychometric properties [51, 52].

### Physiological measures

Physiological activity is continuously recorded throughout the experiment using a personalized wireless BioSignalsPlux Explorer portable device at a sampling rate of 1000 Hz, with data acquisition via OpenSignals software (PLUX – Wireless Biosignals S.A., Lisbon, Portugal). SC and HR are recorded throughout all sessions as measures of arousal. SC is measured using two electrodes attached to the proximal area of the participant’s non-dominant palm. HR is recorded by electrocardiography (ECG) acquired with a single-lead differential bipolar ECG sensor connected to three 24 mm disposable Ag–AgCl electrodes (Kendall™ H135SG, Covidien Deutschland GmbH, Germany). Electrode placement is equivalent to standard medical-grade V6 lead. Cortisol levels are measured via saliva at arrival, after the BAT, and immediately after the exposure. Saliva is collected using Salivette tubes (Sarstedt AG, Nümbrecht, Germany). Participants are instructed to gently move the swab around in their mouth for approximately one minute before placing it back into the tube. Samples will be stored at − 20 °C until laboratory analysis. In accordance with the study by Hauck et al. [31], blood glucose levels are measured on arrival, 17 min after glucose or placebo administration, and again after the exposure phase is completed. On the third experimental day, only one measurement at arrival is taken, since no manipulation or exposure occurs. Blood glucose levels are measured with a blood glucose meter (ACCU-Check Instant, 2021, Roche Diabetes Care GmbH, Mannheim, Germany).

#### Measurement of threat expectancy change (TEC)

The individual threat belief is defined at the beginning of session one, alongside the instructed experimenter. Before and after each exposure, a threat expectancy rating (0% *not at all*—100% *maximal anxiety*) is collected. After the glucose administration and exposure, participants complete a threat occurrence rating (did the threat occur and to what percentage, 0%−100%) as well as a rating of maximum of fear experienced during the speaking exercise (SUDS), and an adjusted threat expectancy rating (how likely would the threat be to occur, if the exercise were repeated, 0% *unlikely*—100% *likely*). Both, the absolute and relative expectancy violation will be calculated [53, 54].

### Procedure

The experiment begins with an initial online screening, followed by a pseudorandom assignment of eligible participants to one of two groups (Glucose vs. Placebo) based on order of participation to maintain balanced group sizes. Participants are blinded, as well as experimenters. An online pretest is conducted one week prior to the first laboratory session to establish baseline self-report measures and reduce the duration of experimental days. Three laboratory sessions in total are conducted at the Department of Clinical Psychology and Psychotherapy at Saarland University led by two trained experimenters from a larger team of experimenters. To ensure a comparable glycemic state for the manipulation, all participants are asked to restrain from eating from 10 pm the day before the laboratory sessions [31]. Laboratory testing takes place between 8:00 and 12:00 am to control for daytime effects in glucose metabolism [55]. The experiment follows a spaced design, with a two-day interval between the first and second testing sessions, and a five-day interval between the second and third sessions, similar to Cheng and colleagues (2017) [11]. Four weeks after the final laboratory session, participants complete an online follow-up. Figure 2 provides an overview of the study procedure.

### Screening

Initial contact is established via e-mail, after which participants arrange an online screening session with one of the experimenters. This screening consists of maximum 30 min of inclusion and exclusion questions, including a brief evaluation of mental health status.

### Pretest

**Fig. 1:**
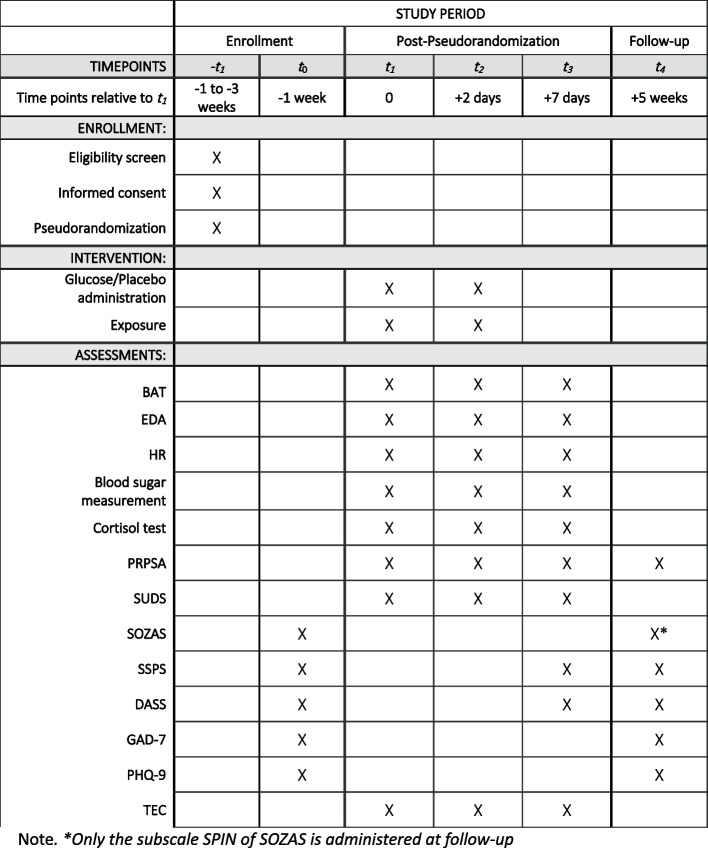
Participant timeline: Schedule of enrollment, interventions, and assessments

Participants receive a link to the pretest via e-mail one week prior to the first laboratory session. The self-report questionnaires are conducted via the SoSci Survey platform [56]. The questionnaires used in the pretest can be found in Fig. 1.

### BAT

The BAT consists of a one-minute speech in front of a neutral audience of three confederates, who are instructed not to react (e.g., no nodding or smiling) and to maintain a neutral expression. Following the SUDS rating, the participant is instructed by an onscreen prompt to stand up and move from behind a partition wall to the front of the room, where the audience is seated temporarily. The topic of the speech appears on a second screen on a desk to the right of the participant. A neutral sound marks the beginning and end of the one-minute speech. After completing the one-minute speech, the participant returns behind the partition wall, and the audience leaves. After a second SUDS rating is administered, a 30-s break follows.

### Exposure phase

**Fig. 2:**
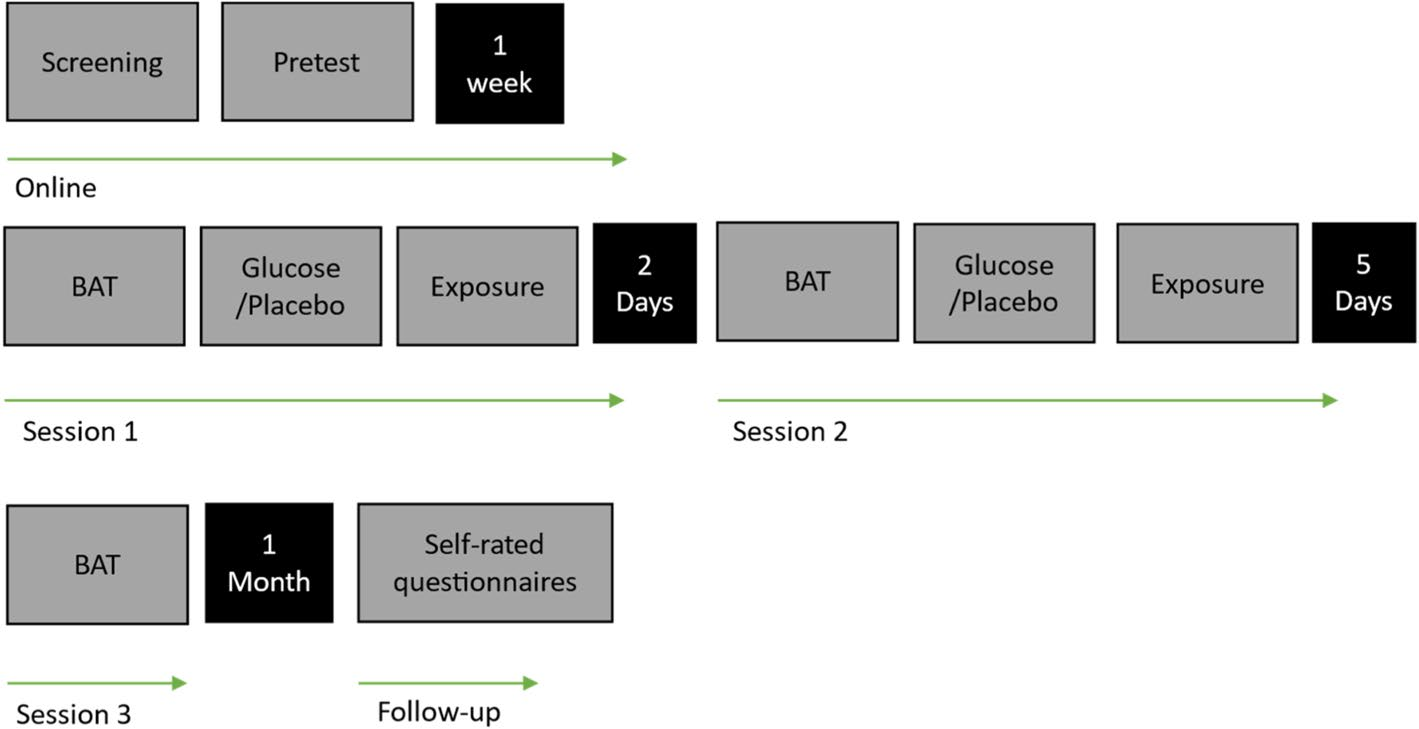
General procedure

In the exposure phase the same sequence of events as in the BAT, that is pre-SUDS followed by a one-minute speech, post-SUDS and break, is repeated eight times in total, with different speech topics for each of the eight speeches. Contrary to the BAT, the audience is instructed to have a friendly expression and react with smiling and nodding if appropriate, in order to provide a positive experience for the participant. The exposure procedure takes place in laboratory sessions 1 and 2 (Fig. 2).

### Audience

Two different audiences of three confederates are present for the speech tasks, for both BAT and exposure. After each exposure phase, every confederate is asked to fill in a speech rating in a separate room from the participant. To avoid familiarity effects, no confederate takes part in both the BAT and the exposure phases for any given participant. Consequently, a total of 15 confederates is required per participant. The confederate pool varies over time with confederates being recruited through flyers, distributed at the university and in the surrounding area, as well as students participating as part of a study course or internship.

### Laboratory sessions

**Fig. 3:**
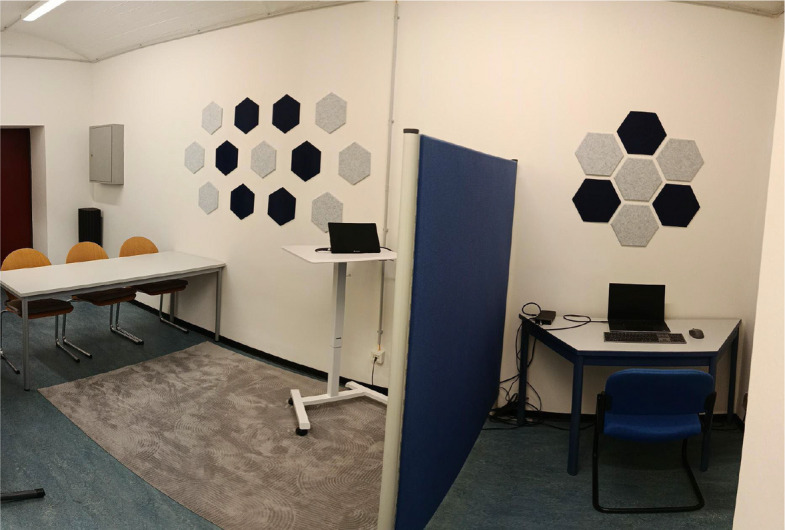
Laboratory setting

The laboratory sessions take place in a specially designed laboratory at Saarland University, depicted in Fig. 3. All laboratory sessions start in the same way: participants are attached to the wireless BioSignalsPlux Explorer portable device (PLUX – Wireless Biosignals S.A.). Saliva cortisol collection and blood glucose measurement are explained at the beginning of session 1 and conducted at the predefined time points (see physiological measures). The experiment is conducted via E-Prime (Psychology Software Tools, Inc., 2016) and starts with a three-minute resting phase, followed by the BAT and a second resting phase. For sessions one and two, a glucose or placebo drink is administered then. To ensure the correct amount of glucose in the bloodstream, a 20-min waiting period follows [31]. During this waiting period, the individual threat belief for the TEC is defined (day 1). Afterwards the exposure phase begins followed by a final three-minute resting phase, that concludes the sessions. Since there is no glucose or placebo administration and exposure at session three, the session ends after the second resting phase.

### Follow up

An automatic email is prepared to send the link to the post-test four weeks after the last laboratory session. The instruments applied can be found in Fig. 1.

### Data collection and statistical analysis

#### Outcome measures

The primary outcome measure is self-reported anxiety (SUDS) during BATs and exposure, as well as PRPSA during BAT, measured on sessions 1, 2, and 3. Secondary outcome measures are the physiological responses SC and HR. Exploratory heart rate variability (HRV) measures will be derived from the ECG signal. As an exploratory biomarker of stress physiology, salivary cortisol will be assessed given its relevance for exposure outcomes and its close link to metabolic and stress responses. Tests will be one-tailed for directional hypotheses and two-tailed when no directional hypothesis is specified, with an alpha level set at 0.05.

#### Treatment of physiological data

R-peaks in the ECG signal are automatically identified and subsequently reviewed manually in ANSLAB (Autonomic Nervous System Laboratory, University of Basel, Switzerland; [57]) to correct for artifacts such as false detections or missed beats. From the cleaned ECG, successive interbeat intervals (IBIs) are derived, which are then used to compute instantaneous heart rate (HR). Time- and frequency-domain indices of HRV will additionally be calculated in ANSLAB. Specifically, the root mean square of successive differences of IBIs (RMSSD) and log-transformed high-frequency power (HF-HRV) during time frames of interest will be extracted for exploratory analysis. SC will be visually edited for artifacts and non-specific skin conductance responses, as well as skin conductance level will be extracted via ANSLAB (Autonomic Nervous System Laboratory, University of Basel, Switzerland; [57]).

### Analysis

To test the main hypothesis, an interaction effect, several mixed-design ANOVAs with the between-participants factor Group (glucose vs. placebo) and the within-participants factor Time (SUDS-BAT/PRPSA on session 1 vs. 2 vs. 3; SUDS-BAT vs First Half of Exposure vs Last Half of Exposure on session 1; SUDS-First Half of Exposure vs Second Half of Exposure vs BAT on session 2) will be calculated.

## Discussion

We present a protocol to investigate potential enhancing effects of glucose administration on an exposure procedure in a sub-clinical sample. A substantial number of individuals fail to achieve clinically significant symptom relief from exposure-based therapies or experience a return of fear following exposure therapy [6, 58]. Anxious individuals show deficits in the mechanisms believed to be central to extinction learning, and such deficits may not only contribute to poor response to exposure therapy but also to the development of excessive fear and anxiety in the first place [13, 15, 59]. Given that exposure-based therapy still has room for improvement, research into an easy-to-apply, low-side-effect, and economical substance like glucose is of great importance. As such, there is tremendous clinical value in enhancing treatment efficacy in general. Basic research plays a crucial role in advancing our understanding of the mechanisms underlying fear learning, as well as identifying ways in which these processes can be modulated. However, it is equally important to take further steps toward translating these findings into clinical practice. Protocols such as the one presented here represent a critical step in this direction: (sub)clinical samples and standardized procedures provide a valuable bridge between laboratory findings and clinical applications. This staged approach is essential, as fully clinical studies are often more expensive and time-consuming. Ultimately, such translational work has the potential to improve therapeutic outcomes. In addition, the standardized structure of this protocol enables straightforward replication and adaptation to other experimental manipulations, thereby ensuring the comparability of results across studies. This facilitates the systematic continuation of research on potential adjuvants for anxiety disorders and supports cumulative progress in translational psychophysiology.

### Trial status

First, a pilot phase was done, to ensure that technical issues were solved and the procedure would work with no limitations. The current trial status is 16 (11♀) participants in total. In total 59 participants were screened, 41 had to be excluded due to not fulfilling inclusion criteria and 2 did not attend the first laboratory session. The experiment will continue running until a total of 76 participants is reached. The expected complete date of the recruitment is June 2027. 

### Strengths and limitations

This study protocol adheres to the principles of open and reproducible science in the form of a randomized, controlled trial. The implemented exposure treatment is well-established and standardized. It fosters the ongoing development of research with detailed information enabling researchers to replicate as well as increasing transparency and therefore expanding open science. This is the first study to investigate the effects of glucose in a (sub)clinical population by translating results from fear conditioning experiments into clinical context. Due to the spaced design, the study faces an extended experimental phase and increased participant dropout. Additionally, the in vivo speech exposure requires a large pool of confederates to provide a live audience, resulting in significant organizational effort.

## Supplementary Information

Supplementary Material 1.

## Data Availability

The data collected within this study are recorded, stored and evaluated in a pseudo-anonymous fashion. A corresponding list with the participant code and name is kept under confidentiality standards until the final completion of the data collection. This data is then destroyed for subsequent full anonymization. The raw physiological data is recorded in an anonymous fashion. In order to ensure good scientific practice, the experimental plans and hypotheses were pre-registered before the experiments started and data is collected (German trial register, https://www.drks.de/drks_web/). In the context of publication, the anonymized raw data will be made available in a data repository for subsequent scientific use. The evaluation of the acquired data is carried out exclusively for scientific purposes.

## Abbreviations

ANOVA: Analysis of Variance
BAT: Behavioral Approach Test
BMI: Body mass index
CBT: Cognitive Behavioral Therapy
DASS: Depression Anxiety Stress Scale
ECG: Electrocardiography
GAD: Generalized anxiety disorder
HR: Heart Rate
PHQ: Patient Health Questionnaire
PRPSA: Personal Report of Public Speaking Anxiety
PSA: Public Speaking Anxiety
SOZAS: Scales for Social Anxiety Disorders
SSPS: Self Statements during Public Speaking Scale
SC: Skin Conductance
SAD: Social Anxiety Disorder
SIAS: Social Interaction Anxiety Scale
SPIN: Social Phobia Inventory
SPS: Social Phobia Scale
SUDS: Subjective Units of Disturbance Scale
