# Supplementary material for: Sweetening fear exposure: study protocol for a multi-day, randomized controlled trial to study effects of glucose in exposure sessions of participants with public speaking anxiety
Source: BMC Psychol. 2026 Feb 18;14:268. doi: 10.1186/s40359-026-04125-0 (PMC12933903; doi:10.1186/s40359-026-04125-0)
Supplement: Supplementary file 1 — Supplementary Material 1. [file 40359_2026_4125_MOESM1_ESM.docx]

Supplementary file 1 additional questionnaires

The following section presents questionnaires that are not directly relevant to the main hypotheses of the current project. These questionnaires are administered at the time points shown in Fig. S1 as part of research-based teaching projects.

Anxiety Change Expectancy Scale (ACES)

The ACES (Dozois & Westra, 2005) is a 20-item measure assessing the anticipation of anxiety change. Items are rated on a 5-point Likert scale (1 = *strongly disagree* to 5 = *strongly agree*). Total scores range from 20 to 100, with higher scores indicating more positive expectations of change.

Anxiety Sensitivity Index-3 (ASI-3)

The ASI-3 (Taylor et al., 2003; German version: Kemper et al., 2009) includes 18 items assessing anxiety sensitivity, i.e., fear of symptoms related to sympathetic activation. Items are rated on a five-point scale (0 = *strongly disagree* to 4 = *strongly agree*). The scale comprises three subscales with six items each: Somatic, Social, and Cognitive Concerns. The total score ranges from 0 to 72. The inclusion of the ASI-3 complements physiological and other self-report measures by assessing cognitive–affective interpretations of bodily sensations, thereby providing insight into subjective anxiety processing that is not accessible through physiological indicators alone.

Experiential Avoidance Rating Scale (EARS)

The EARS (Borgogna et al., 2023; German version: Ferreira de Sá et al., 2025) is a six-item self-report measure of experiential avoidance, rated on a five-point scale ranging from 1 (*never true*) to 5 (*very often true*). Higher total scores indicate greater levels of experiential avoidance. The EARS has good to excellent psychometric properties. The scale provides additional insight by measuring actual avoidance behaviors, offering a behavioral counterpart to cognitive and physiological assessments.

Big Five Inventory – 10 (BFI-10)

The BFI-10 (Rammstedt et al., 2013) is the German short version of the Big Five Inventory that assesses the five major personality dimensions (extraversion, agreeableness, conscientiousness, neuroticism, and openness) with 10 Items. All items are rated on a five-point Likert scale (1 = *disagree strongly* to 5 = *agree strongly*).

Perceived Stress Scale (PSS)

The PSS (Cohen et al., 1983; German version: Klein et al., 2016) measures perceived stress via ten items on a 5-point Likert scale from 1 (*never*) to 5 (*very often*). It includes the subscales Helplessness (6 items) and Self-efficacy (4 items). Total scores range from 10 to 50, with higher values reflecting greater stress. The instrument demonstrates adequate psychometric quality (Klein et al., 2016; Lee, 2012).

Intolerance of Uncertainty (UI-18)

The UI-18, the short version of the Intolerance of Uncertainty Scale (Freeston et al., 1994; German version: Gerlach et al., 2008), measuring intolerance of uncertainty trough 18 items rated from 1 (*not characteristic of me at all*) to 5 (*very characteristic of me*). It includes three subscales: Restricted Ability to Act, Burden and Vigilance, each consisting of six items. Total scores range from 18 to 90, with higher scores indicating greater intolerance of uncertainty. The construct is linked to the development and maintenance of anxiety disorders (Morriss et al., 2016).

Sense of Coherence (SOC L9)

The SOC L9 is the Leipzig Short Scale of the Sense of Coherence Scale (Antonovsky, 1993; German version: Schumacher et al., 2000). It consists of 9 items and is measured on a scale ranging from 1 to 7 with item specific verbal anchors. The total score ranges from 9 to 63, and higher scores represent a stronger sense of coherence.

Brief Resilience Scale (BRS)

The BRS (Smith et al., 2008; German version: Chmitorz et al., 2018; Kunzler et al., 2018) measures resilience using six items rated on a five-point Likert scale from 1 (*strongly disagree*) to 5 (*strongly agree*). The results are based on the mean value of all items. The instrument shows good psychometric properties in both healthy and clinical settings (Broll et al., 2024; Chmitorz et al., 2018).

Credibility Expectancy Questionnaire (CEQ)

The CEQ (Devilly & Borkovec, 2000) is a short scale used to measure treatment expectancy and rationale credibility, designed to be used in clinical settings. In total six items are used with item specific verbal anchors. Half of the items assess expectancy, while the other half measure credibility of the treatment.

Modified Perception of Speech Performance (MPSP)

The MPSP (Cody & Teachman, 2011) measures the perceived speech performance. It consists of 12 Items on a 5-point scale ranging from 0 (*not at all*) to 4 (*very much*). During this experiment, the rating is administered to the participants as self-rated measure, as well as to the audience as objective measure of speech performance.

Childhood Trauma Questionnaire CTQ

The CTQ (Bernstein et al., 2003; German version: Klinitzke et al., 2012) is a short scale consisting of 28 items on a five-point Likert scale ranging from 1 *(not at all*) to 5 (*very often*). The questionnaire comprises five subscales (emotional abuse, physical abuse, sexual abuse, emotional neglect, and physical neglect).

Personal Attributes Questionnaire (PAQ-8)

The PAQ-8 is a short version based on the PAQ (Spence et al., 1975; German version GEPAQ: Tibubos et al., 2022). Eight Items are rated on a five-point scale, with endpoints anchored by gender expression labels indicating the degree to which the statement applied. A mean is formed from the four items for the masculine and the feminine subscales. Higher values indicate a stronger expression of attributes.

Patient Global Impression of Improvement (PGI)

The PGI (Bjelic-Radisic, 2013; German version: Yalcin & Bump, 2003) consists of one Item rated on a Scale from 1 (*very much better*) to 7 (*very much worse*). It measures subjective improvement after treatment.

Anxiety and Arousal

Two questions on Anxiety and Arousal on a visual analogue scale (0%-100%) are being asked at arrival of the present study. (Anxiety: “How anxious do you feel right now?”; Arousal: “How alert do you feel right now?”)

**Fig. S1**

Participant timeline: Schedule of enrollment, interventions, assessments and additional assessments.

|  | **STUDY PERIOD** | | | | | |
| --- | --- | --- | --- | --- | --- | --- |
|  | **Enrollment** | | **Post-pseudorandomization** | | | **Follow-up** |
| **TIMEPOINTS** | ***-t_1_*** | ***t*_0_** | ***t_1_*** | ***t_2_*** | ***t_3_*** | ***t_4_*** |
| **Time points relative to *t_1_*** | **-1 to -3 weeks** | **-1 week** | **0** | **+2 days** | **+7 days** | **+5 weeks** |
| **ENROLLMENT:** |  | | | | | |
| **Eligibility screen** | X |  |  |  |  |  |
| **Informed consent** | X |  | X |  |  |  |
| **Pseudorandomization** | X |  |  |  |  |  |
| **INTERVENTION:** |  | | | | | |
| **Glucose/Placebo**  **administration** |  |  | X | X |  |  |
| **Exposure** |  |  | X | X |  |  |
| **ASSESSMENTS:** |  | | | | | |
| **BAT** |  |  | X | X | X |  |
| **EDA** |  |  | X | X | X |  |
| **HR** |  |  | X | X | X |  |
| **Blood sugar measurement** |  |  | X | X | X |  |
| **Cortisol test** |  |  | X | X | X |  |
| **PRPSA** |  |  | X | X | X | X |
| **SUDS** |  |  | X | X | X |  |
| **SOZAS** |  | X |  |  |  | X* |
| **SSPS** |  | X |  |  | X | X |
| **DASS** |  | X |  |  | X | X |
| **GAD-7** |  | X |  |  |  | X |
| **PHQ-9** |  | X |  |  |  | X |
| **TEC** |  |  | X | X | X |  |
| **ADDITIONAL**  **ASSESSMENTS** |  | | | | | |
| **ACES** |  | X |  |  | X | X |
| **ASI-3** |  | X |  |  |  | X |
| **EARS** |  | X |  |  | X | X |
| **BFI** |  | X |  |  |  | X |
| **PSS** |  | X |  |  |  | X |
| **UI-18** |  | X |  |  |  | X |
| **SOC-L9** |  | X |  |  |  | X |
| **BRS** |  | X |  |  |  | X |
| **CEQ** |  |  |  |  | X |  |
| **MPSP** |  |  | X | X |  |  |
| **CTQ** |  | X |  |  |  | X |
| **PAQ-8** |  | X |  |  |  |  |
| **PGI** |  |  |  |  |  | X |
| **Anxiety and Arousal** |  |  | X | X | X |  |

Note*. *Only the subscale SPIN of SOZAS is administered at follow-up*
